# Resource-Stratified Guidelines for Cancer Management: Correction and Commentary

**DOI:** 10.1200/JGO.2016.006213

**Published:** 2016-08-31

**Authors:** Benjamin O. Anderson, Catherine Duggan

**Affiliations:** **Benjamin O. Anderson**, Fred Hutchinson Cancer Research Center and University of Washington, Seattle, WA; **Catherine Duggan**, Fred Hutchinson Cancer Research Center, Seattle, WA

## To the Editor:

The article by Chuang et al,^1^ “Management and Care of Women with Invasive Cervical Cancer: ASCO Resource-Stratified Clinical Practice Guideline,” recently published in *Journal of Global Oncology*, providing evidence-based, resource-stratified global recommendations on the management and palliative care of women diagnosed with invasive cervical cancer, is timely, especially in low- and middle-income countries (LMICs), where cervical cancer prevention and early detection strategies lag behind those in high-income countries. There were an estimated 266,000 deaths from cervical cancer worldwide in 2012, accounting for 7.5% of all female cancer deaths.^2^ Almost nine out of 10 (87%) of cervical cancer deaths occur in the less-developed regions of the world, making the use of resource-stratification methodology highly appropriate for providing optimal recommendations that have the potential to be applied in real-world settings.

On behalf of the Breast Health Global Initiative (BHGI) and the more than 300 multidisciplinary experts involved in its guideline development, we wish to respectfully inform the ASCO Consensus Panel authors that they have incorrectly attributed their resource-stratification methodology to the World Health Organization (WHO) rather than to BHGI. They have also incorrectly cited the peer-review publications describing the concepts of resource stratification that they have applied to the management of invasive cervical cancer. This work should properly be attributed to BHGI, which was the first to develop, test, and validate the concept of a four-tiered resource stratification framework—basic, limited, enhanced, and maximal resource levels—in which cancer management strategies can be prioritized within the context of available health care resources. The purpose of this brief review is to recount the history and correct the citations for current and future ASCO resource-stratified guidelines.

In 2002, WHO published an executive summary of their National Cancer Control Programs Policies and Managerial Guidelines.^3^ In this monograph, WHO described three country resource scenarios (low, medium, high) and suggested that actions by national cancer control programs for cancer early detection, diagnosis, treatment, and palliation should be prioritized based on available resources (Table 1 of their publication). WHO did not suggest any approach or mechanism to guide evidence-based cancer control planning, nor did WHO develop the four-tiered resource stratification framework as suggested by Chuang et al.^1^

Between 2002 and 2013, and through a series of five collaborative global summits, BHGI developed a resource-stratified framework to guide all aspects of breast cancer management. Although WHO participated in BHGI’s Global Summits and in some cases contributed as coauthors to BHGI’s publications, it was not until 2012 that BHGI was first invited to present the resource-stratified guideline model at WHO headquarters in Geneva. WHO subsequently asked BHGI to provide technical advice at their Strategic Technical Meeting on Management of Cancer (April 2015) and the WHO Steering Committee for Selection and Classification of Medical Devices for Cancer Management (September 2015). However, the WHO Guideline Review Committee has to date not developed, adopted, or endorsed this resource-stratified guideline approach for cancer control or management. To this end, we applaud ASCO for using these guidelines as a framework for management of invasive cervical cancer in different resource settings. We suggest that they be offered to WHO as further evidence that resource stratification is a valuable and important tool for translating evidence into action on the basis of a realistic assessment of existing in-country health care resources.

BHGI held its first Global Summit in October 2002 in Seattle, Washington and invited global experts to address global disparities in breast cancer early detection, diagnosis, and treatment. The core findings of this summit were published as a supplement to *The Breast Journal*^3A^ and provided the basis of BHGI’s subsequent guideline work over the next 12 years. In January 2005, BHGI first presented its resource-stratification methodology at the second BHGI Global Summit in Bethesda, Maryland hosted by the National Cancer Institute Office of International Affairs. This 2005 Global Summit brought together more than 60 international experts from 33 countries representing all geographic regions and economic levels. These experts came from diverse specialties related to breast cancer management, including radiology, pathology and cytology, surgery, oncology, radiation therapy, health economics, medical ethics, sociology, and patient advocacy. This group examined existing evidence on breast cancer early detection, diagnosis, and treatment and subsequently published the second BHGI supplement in *The Breast Journal*^3B^ presenting these results. The 2006 supplement’s overview article^4^ presented for the first time the four-tier resource-stratification framework (basic, limited, enhanced, maximal) that has now been applied by the ASCO Consensus Panel. In that same supplement, four-tier resource-stratified guidelines were published for breast cancer early detection,^5^ diagnosis and pathology,^6^ treatment,^7^ and health care systems.^8^ As presented in the 2006 methodology overview,^4^ BHGI used a well-defined and evidence-organized approach (primary literature review, evidence examination, structured multidisciplinary consensus development, team authorship, external peer review, and final publication). As original sources for the resource-stratified guideline methodology, these 2006 publications would be most appropriate to reference as the initial and foundational citations. We note that the ASCO Consensus Panel used a similar methodology—a modified Delphi formal expert consensus approach that was also informed by ADAPTE. We applaud ASCO’s rigorous methods, which we believe nicely mirror the approach first applied by BHGI 10 years previously.

Of historic note, ASCO itself played a key organizational role in the subsequent reexamination and revision of the BHGI resource-stratified guidelines in BHGI’s third guideline cycle, from 2007 to 2008. BHGI organized its third Global Summit to review, analyze, and validate the four-tier resource-stratification findings created by the previous 2005 BHGI panels. ASCO was the host organization for that 2007 Global Summit, providing a generous unrestricted educational grant to hold the meeting from October 1 to 4, 2007 in Budapest, Hungary. Gabriel N. Hortobágyi, MD, who was both ASCO’s immediate past president and BHGI’s executive committee chair, welcomed 100 breast cancer and global health experts from 40 countries who had gathered to collaborate in the third cycle of BHGI’s guideline analysis and publication. This new consensus expert group reexamined and validated the prior resource-stratification assignments. To further define a framework for resource-stratified implementation measurement, the BHGI panels created a series of implementation quality metrics (process and outcomes measures) directly linked to the resource-stratification tables. The results of the 2007 BHGI Global Summit were published in 2008 as a supplement to the journal *Cancer*^9^ and included resource-allocation monographs on early detection,^10^ diagnosis,^11^ treatment,^12^ and health care systems.^13^ During this same 2007 summit, BHGI formed multispecialty focus groups to address specific implementation issues in breast pathology,^14^ breast radiation therapy,^15^ and management of locally advanced breast cancer.^16^ As such, ASCO became linked to BHGI and its guideline development process several years before embarking on its own process of resource-stratification guideline development process in cervical cancer.

The BHGI resource-stratified guidelines have been well received in the global health literature.^17^ A systematic literature review and citation analysis performed in 2013 showed that of the 552 unique articles citing the BHGI guidelines, 359 (65%) referenced either the guidelines’ overviews (200 [36%]) or the early detection guidelines (159 [29%]) and the remaining guideline topics with decreasing frequently: breast cancer diagnosis (52 [9%]), treatment (84 [15%]), and the changes needed to the health care systems (48 [9%]). The BHGI health care system guidelines have been increasingly referenced over time, and by 2011, exceeded the articles referencing diagnostic guidelines. Of the 552 documents, 283 (51%) focused on LMICs in sub-Saharan Africa (77 [14%]), Asia (71 [13%]), Latin America (71 [13%]), and the Middle East (50 [9%]). Another 181 (33%) articles were non–country specific, and the remainder came from high-income countries. Overall, 375 (68%) cited BHGI guidelines as a method related to disease management and noted that the guidelines could be applied to develop a country-level course of action in breast cancer management.

At least three other groups have applied or adapted BHGI’s framework in resource-stratified guideline development before the most recent ASCO Consensus Panel:

- At the 2009 Asian Oncology Summit in Singapore, consensus groups were organized by the editorial leadership of *Lancet Oncology* to create and publish resource-stratified guidelines for Asia as related to human epidermal growth factor receptor 2/neu-positive breast cancer,^18^ head and neck cancers,^19^ T-cell and natural killer cell neoplasms,^20^ non–small-cell lung cancer,^21^ hepatocellular carcinoma,^22^ and endometrial cancer.^23^
- In 2015, the World Bank published *Cancer: Disease Control Priorities, Third Edition, Volume 3* (DCP3),^24^ which for the first time presented a complete volume on cancer care in LMICs. DCP3 developed an essential package of potentially cost-effective measures for countries to consider and adapt, including: prevention of tobacco-related cancer and virus-related liver and cervical cancers; diagnosis and treatment of early breast cancer, cervical cancer, and selected childhood cancers; and widespread availability of palliative care, including opioids.^25^ In the economic overview from this seminal publication, DCP3 explicitly applied and acknowledged the BHGI resource-stratification framework for global cancer care in assessing feasibility and likely cost effectiveness of interventions by resource level for breast cancer, cervical cancer, colorectal cancer, liver cancer, oral cancer, and pediatric cancer. On this basis, DCP3 made explicit resource-stratified recommendations for primary prevention, cancer screening and detection, treatment with curative intent, and management of advanced disease.^26^
- Most recently, the National Comprehensive Cancer Network has now created and electronically published its own resource-stratified frameworks^27^ for cervical cancer, breast cancer, gastric cancer, hepatobiliary cancer, non–small-cell lung cancer, and prostate cancer, also on the basis of the BHGI methods and approach.^28^

As a group and partnership, BHGI is gratified that resource-stratification methods are being adopted, adapted, and incorporated by organizations striving to improve cancer outcomes around the globe, and specifically in limited-resource settings. At the same time, we must collectively acknowledge that these efforts represent a beginning to the much larger process of guideline implementation in LMICs. International collaborations are needed to define national cancer control planning to help implement evidence-based findings in realistic health policy changes to improve cancer outcomes. We recommend that the scientific principles of implementation science on the basis of a resource-stratified framework be thoughtfully applied to guide effective strategies for managing cancer around the world. Only then will we begin to see the fruits of our combined labors manifest as improved outcomes for patients with cancer around the globe.

## References

[1] Chuang LT, Temin S, Camacho R, et al: Management and care of women with invasive cervical cancer: American Society of Clinical Oncology resource-stratified clinical practice guideline. J Glob Oncol 10.1200/JGO.2016.003954 10.1200/JGO.2016.003954 PMC5493265 28717717

[2] Ferlay J, Soerjomataram I, Ervik M, et al: GLOBOCAN 2012 v1.0, Cancer Incidence and Mortality Worldwide: IARC CancerBase No. 11. http://globocan.iarc.fr

[3] World Health Organization: Executive Summary of the National Cancer Control Programmes: Policies and Managerial Guidelines. Geneva, World Health Organization, 2002

[3A] Anderson BO, Braun S, Carlson RW, et al: Breast J 9:S39-S74, (suppl 2) [e-pub April 26, 2003]

[3B] Masood S, Anderson BO, Shyyan R, et al: Breast J 12:S1-S122, (suppl 1) [e-pub January 25, 2006]

[4] Anderson BO, Shyyan R, Eniu A (2006). Breast cancer in limited-resource countries: An overview of the Breast Health Global Initiative 2005 guidelines. Breast J.

[5] Smith RA, Caleffi M, Albert US (2006). Breast cancer in limited-resource countries: Early detection and access to care. Breast J.

[6] Shyyan R, Masood S, Badwe RA (2006). Breast cancer in limited-resource countries: Diagnosis and pathology. Breast J.

[7] Eniu A, Carlson RW, Aziz Z (2006). Breast cancer in limited-resource countries: Treatment and allocation of resources. Breast J.

[8] Anderson BO, Yip CH, Ramsey SD (2006). Breast cancer in limited-resource countries: Health care systems and public policy. Breast J.

[9] Anderson BO, Yip CH, Smith RA (2008). Guideline implementation for breast healthcare in low-income and middle-income countries: Overview of the Breast Health Global Initiative Global Summit 2007. Cancer.

[10] Yip CH, Smith RA, Anderson BO (2008). Guideline implementation for breast healthcare in low- and middle-income countries: Early detection resource allocation. Cancer.

[11] Shyyan R, Sener SF, Anderson BO (2008). Guideline implementation for breast healthcare in low- and middle-income countries: Diagnosis resource allocation. Cancer.

[12] Eniu A, Carlson RW, El Saghir NS (2008). Guideline implementation for breast healthcare in low- and middle-income countries: Treatment resource allocation. Cancer.

[13] Harford J, Azavedo E, Fischietto M (2008). Guideline implementation for breast healthcare in low- and middle-income countries: Breast healthcare program resource allocation. Cancer.

[14] Masood S, Vass L, Ibarra JA (2008). Breast pathology guideline implementation in low- and middle-income countries. Cancer.

[15] Bese NS, Munshi A, Budrukkar A (2008). Breast radiation therapy guideline implementation in low- and middle-income countries. Cancer.

[16] El Saghir NS, Eniu A, Carlson RW (2008). Locally advanced breast cancer: Treatment guideline implementation with particular attention to low- and middle-income countries. Cancer.

[17] Echavarria MI, Anderson BO, Duggan C (2014). Global uptake of BHGI guidelines for breast cancer. Lancet Oncol.

[18] Wong NS, Anderson BO, Khoo KS (2009). Management of HER2-positive breast cancer in Asia: Consensus statement from the Asian Oncology Summit 2009. Lancet Oncol.

[19] Wee JT, Anderson BO, Corry J (2009). Management of the neck after chemoradiotherapy for head and neck cancers in Asia: Consensus statement from the Asian Oncology Summit 2009. Lancet Oncol.

[20] Kwong YL, Anderson BO, Advani R (2009). Management of T-cell and natural-killer-cell neoplasms in Asia: Consensus statement from the Asian Oncology Summit 2009. Lancet Oncol.

[21] Soo RA, Anderson BO, Cho BC (2009). First-line systemic treatment of advanced stage non-small-cell lung cancer in Asia: Consensus statement from the Asian Oncology Summit 2009. Lancet Oncol.

[22] Poon D, Anderson BO, Chen LT (2009). Management of hepatocellular carcinoma in Asia: Consensus statement from the Asian Oncology Summit 2009. Lancet Oncol.

[23] Tangjitgamol S, Anderson BO, See HT (2009). Management of endometrial cancer in Asia: Consensus statement from the Asian Oncology Summit 2009. Lancet Oncol.

[24] Gelband H, Jha P, Sankaranarayanan R, et al: (eds). Cancer: Disease Control Priorities (ed 3). Washington, DC, 2015 26913318

[25] Gelband H, Sankaranarayanan R, Gauvreau CL, et al: Costs, affordability, and feasibility of an essential package of cancer control interventions in low-income and middle-income countries: Key messages from Disease Control Priorities, 3rd edition. Lancet 387:2133-2144, 2016 10.1016/S0140-6736(15)00755-2 26578033

[26] The World Bank: Cancer in low and middle-income countries: An economic overview, in Gelband H, Jha P, Sankaranarayan R (eds). Disease Control Priorities in Developing Countries (ed 3). Washington, DC, 2015 26913333

[27] National Comprehensive Cancer Network: NCCN Framework for Resource Stratification of NCCN Guidelines (NCCN Framework). https://www.nccn.org/framework/10.6004/jnccn.2016.0103 27496112

[28] Carlson RW, Scavone J, Koh W-J (2016). NCCN Framework for resource stratification: A framework for providing and improving global quality oncology care. J Natl Compr Canc Netw.

